# Supplementary material for: Regulatory scripting: Stakeholder participation in food and drug administration medical device advisory meetings
Source: PLoS One. 2023 Feb 16;18(2):e0281774. doi: 10.1371/journal.pone.0281774 (PMC9934387; doi:10.1371/journal.pone.0281774)
Supplement: Attachment — Submitted filename: Response to Reviewers.docx [file pone.0281774.s001.docx]

**Review Comments to the Author (Responses in red font)**

Reviewer #1: Thank you very much for the interesting manuscript. I would suggest you consider deleting the table describing the number of panel meetings of each panel as this appears peripheral to your main points. More importantly, your most interesting findings are your nuanced analysis of the role of patients and non-physician advocates and the type and tenor of comments they are likely to make. You may wish to increase your focus on this area and summarize your findings at the end of your manuscript. There is a lot of interesting information in this manuscript, but it may be more powerful if it is slightly more focused on the qualitative analyses that you have performed so nicely.

Thank you for these comments. We removed Table 1 from the manuscript and added additional qualitative findings and discussion.

Reviewer #2: This manuscript provides quantitative and qualitative evidence on stakeholder participation in FDA medical device advisory committee meetings. The study is motivated by the fact that there have been ongoing calls to increase patient/public participation in the health policy making process. Because advisory committees are one forum where a broad range of stakeholders can share views with the FDA (via its external advisory committees) they offer, as the authors put it, “one of the few places where patients might have a voice in the regulatory process.”

I share the authors’ view that advisory committees are an interesting site in which to study public engagement in health policy making. I also believe that the technical aspects of this study—sample selection, statistical analysis, qualitative analysis—are well done and the results are clearly presented. However, I think the authors make a questionable choice with respect to a key aspect of the study design that renders the quantitative findings difficult to interpret.

As the authors rightly note (page 6) the open public comment period makes up only a small part of the advisory committee meeting. Thus, it’s unsurprising that comments of public speakers (patients, advocates, etc.) take up a relatively small part of the overall speaking time at the meeting. The authors do note that committee members, and chairs in particular, have some discretion to alter speaking times through follow up questions. But a priori, it seems unlikely that questions would be so extensive as to shift the balance of which groups speak most at meetings, and indeed, the findings bear this out. On their own, then, I don’t think the quantitative findings are particularly compelling. They suggest that speaking times reflect the established structure of advisory committee meetings. Nonetheless the findings may be worth consideration for publication in in this journal given its aims.

We appreciate this comment, and agree that the quantitative findings should not stand alone. We chose to present a mixed method analysis to consider *both* the extent of and the content of the participation of various stakeholders in these FDA hearings. While the quantitative findings do reflect the structure that is provided for FDA stakeholder participation, we feel it is important to document the patterns of participation across stakeholders in this regulatory context, not just in terms of speaking time but also the kinds of evidence stakeholders used and the recommendations that stakeholders made

In response to this comment and those by Reviewer 1, we have reduced the focus on the quantitative findings and expanded the focus on the qualitative findings in the manuscript.

But I think there is a larger challenge in the way the authors interpret the quantitative findings to draw broader conclusions about the weight or value given to patient voice in regulatory decision making. Some representative passages:

“Thus, in addition to an asymmetry whereby industry may have a stronger motivation to participate in policy-making than other stakeholders, the structure seems to reinforce this asymmetry, providing industry greater opportunity than some other parties.”

“While this study did not focus on the impacts of public participation, it documents the variable extent of, and content of, constituent participation – at least one important aspect of public participation. Morone and Kilbreth warn of the potential danger of advisory processes simply legitimating official decisions, without creating true participation.”

“FDA advisory meetings are therefore important loci, one of the few places where patients might have a voice in the regulatory process, though as we have demonstrated, they enjoy a limited hearing based on the current script. Advisory meetings constitute what Anspach calls an ‘ecology of knowledge’, and should be subject to further critical analysis and cross-case comparison.”

I think there are two issues with the interpretation suggested by these and other passages, namely, that the discrepancies in speaking time suggest a lack of regard for patient voice. First, while advisory committee meetings do contain open public hearings, their primary function is not as a forum for public engagement. According to FDA, the “primary role” of the FDA advisory committees is to “provide independent expert advice to the Agency in its evaluation of these regulated products and help the agency move toward making sound decisions based upon reasonable application of sound scientific principles.” (<https://www.fda.gov/patients/about-office-patient-affairs/learn-about-fda-advisory-committees>). It thus seems justifiable that a larger part of the meeting is given over to representatives of the sponsoring company presenting their data and FDA representative presenting the advisory committee with their interpretation of the data. To suggest that the relatively small amount of time given over to public comments is a problem, the authors should do more to explain why it is plausible to think that the public should play a larger role in what are often quite technical discussions.

Thank you for noting these concerns. We have reviewed the manuscript carefully, revised the interpretation of our findings, removed some of the passages noted, and included more qualitative data to better balance the tone of the article. We have otherwise tied the findings to prior research on these topics.

The FDA webpage noted by the Reviewer describes the “activities and operations of federal advisory committees” as having the following three purposes:

- obtain advice from experts who work outside of the government.
- work towards an open and transparent government.
- encourages patients, healthcare providers and other interested people to share their views during the open public hearing or by submitting comments to the open docket.

While the primary purpose of Advisory Committees is described as Reviewer 2 noted, it is clear throughout the FDA website that these committees serve a purpose of engaging the public, which includes patients and family members, advocates, concerned physicians, and other interested parties. In our paper, we aim to assess the processes and dynamics around this particular objective of the FDA (bullet #3 above), in balance of its other stated purposes.

In response to this comment, we have more carefully delineated the purposes of FDA Advisory Committees in our manuscript and described our paper’s purpose of analyzing the extent and content of stakeholder public participation. We appreciate the feedback and the opportunity to more carefully frame these findings and their implications.

Second, advisory committee meetings are by no means the only opportunity for patient engagement at the FDA (<https://www.fda.gov/patients/learn-about-fda-patient-engagement>). Indeed, as the authors note, given the extent to which patients who speak at advisory committee meetings often have financial ties to sponsoring companies, it is not clear that they are a particularly good forums for anything approaching representative public input on FDA decision making. Other initiatives, like the FDA’s more rigorous program for incorporating patient experience data in regulatory decision making (<https://www.fda.gov/drugs/development-approval-process-drugs/assessment-use-patient-experience-data-regulatory-decision-making>) seem a far better way to incorporate patient voice. Overall, then, I find the authors’ interpretation of their quantitative findings to be somewhat misleading.

Though I recognize that it would entail a significant revision of the paper, I might suggest dropping the quantitative findings and focusing instead on the qualitative findings.

We understand that there are additional avenues for patient participation within the FDA. Advisory committee meetings remain an important venue, particularly when there are safety concerns about devices but this comment spurred us to feature the other avenues more carefully in the discussion section of our paper. The first resource link provided by Reviewer 2 outlines the various opportunities, and we have added more context about these opportunities into the manuscript, noting that some represent recent developments within the FDA (for instance, the Patient Engagement Advisory Committee, the only space specifically designated for public participation on medical devices, was first proposed in 2015). The second resource link provided by Reviewer 2 points to the robust process for patient participation in *drug development specifically*. This points to a perennial issue with FDA regulatory processes and public understanding – there are more robust regulatory processes for pharmaceutical drugs than there are for medical devices, though people often assume that these regulatory processes are equivalent (something discussed by Ingrid Nygaard, now added to the manuscript). This underlines why we have focused our analysis on medical devices specifically, and opportunities for public/stakeholder participation therein. We have added a bit more context to the manuscript to explain the unique regulatory concerns with medical device regulation, and noted that these have received less attention than pharmaceutical regulation, referring to work by Alex Faulkner, who describes medical devices as the “Cinderella” of medical technologies. We appreciate this comment because it allowed us to draw out the more limited venues for feedback about medical devices more explicitly.

The Reviewer also notes that patients may have financial ties to sponsoring companies, and thereby may not present representative public input. We have intentionally separated patients (as well as advocates, physicians, and researchers) who were unsponsored by industry from those who were sponsored by industry, to deal with these potential conflicts of interest influencing testimonies. Indeed, the Reviewer’s point speaks to why we made this decision in our coding of participants.

We wish to thank the reviewers sincerely for their thoughtful suggestions, which have strengthened the paper and sharpened what we believe are its central contributions.
